# American Academy of Dental Science

**Published:** 1899-03

**Authors:** 

**Affiliations:** American Academy of Dental Science


### Reports of Society Meetings.

### AMERICAN ACADEMY OF DENTAL SCIENCE.

The regular monthly meeting of the American Academy of Dental Science was held at Young's Hotel, Wednesday evening, October 5, 1898, at six o'clock.

*President Cooke.*—We have with us as guest this evening a gentleman who has contributed very materially in the past to the success and interest of this Academy. I am pleased to introduce to you Dr. Horatio C. Meriam, of Salem, Mass., who will speak to us upon "The Meriam Extension Crown," extra tough gold solder, and annealing steel.

*Dr. Meriam.*—Mr. President and Gentlemen, the chairman of your Executive Committee wrote me a year ago asking if I had anything for the Academy. I had nothing at the time, but later on some things seemed worth reporting, so last spring I wrote him for a night, and he kindly placed this evening at my disposal.

I was troubled to know what to call my report, but finally decided to let it go on the card as you received it.

(For Dr. Meriam's paper, see page 141.)

### DISCUSSION.

*President Cooke.*—I am sure we all feel like thanking Dr. Meriam for the information he has given us. The question is now open for discussion, or rather the three subjects,—the matter of the extension crown, which seems to take the place of a bridge; the question of the gold solder, and the question of annealing steel.

*Dr. Eames.*—I saw only one of the casts that was being passed around, and that was the extension crown. I would like to ask Dr. Meriam in regard to the length of time he has tested this in use, and if, as it appears, the bicuspid rests on the gum tissue, and if it is easily maintained in a cleanly condition.

*Dr. Meriam.*—Entirely so, because it has a bell back; it is set in a cup that has a bell back, and while one rests against the gum tissue, it is no longer than the teeth at either side of it, so that it is not forced up. I have no more expectation of trouble from it than I would from an ordinary ring on a lady's finger, and I think the principle can be carried farther. I think the mouth will tolerate a small gold wire that rests firmly on the membrane, as long as that wire is not irritant and is not movable. When we see what the mouth will tolerate in the way of plates, we are justified in believing that an appliance of this kind will not cause trouble until we see it, and you will see that all parts can be reached with the brush. It is not only clean, but it is bright in the mouth, and I think it has been there about three years.

*Dr. Smith.*—I simply wanted to ask Dr. Meriam about what I did not quite understand in the description of this bridge. I understood him to say one of the bicuspids is not attached to a root.

*Dr. Meriam.*—Neither of them is attached, though there is a root in one case. The end of the root is filled with gutta-percha, and the crown, having a cup or ring at its base, rests against it. On the other side of the cast you will see the same principle carried out, except that in that case there is a loss of the alveolus which is protected by this cup.

*Dr. Smith.*—And the porcelain takes the place of that loss?

*Dr. Meriam.*—No; merely rests against it. The second molar carries the tooth, and there is very little pressure on the gum.

With reference to the model which you see here, something like sixteen years ago this lady came wishing to have all her teeth out. I had a talk with her, and I have had the care of her teeth since. I did an operation of this kind for a lady patient about five years ago, and when I sent my modest bill the check came back with a note,—"If you had charged me one thousand dollars, I should have called it cheap, but I never could have paid you." This case has a band put on that is supported by the buccal and palatal walls only, the tooth being badly decayed; the tooth was then filled with amalgam. This case I had to repair: I simply sawed the band in two, removed it, repaired the case, soldered the band together, and replaced it.

*Dr. Werner.*—Is the band on the molar which you first referred to cemented on?

*Dr. Meriam.*—No, with gutta-percha and oil; but both teeth are conical, and it can be forced up tightly.

*Dr. Werner.*—The ordinary filling gutta-percha softened with oil?

*Dr. Meriam.*—Yes; softened with the oil of sassafras, which is rather pleasanter to the taste than some of the others.

*Dr. Werner.*—I would like to ask for information, how much articulation,—how much force,—do those cases, in your experience, bear well?

*Dr. Meriam.*—Only their share, the normal force required of a tooth in a certain position. You will see the force with which the patient eats, from the fact that all the incisors are worn.

*Dr. Banfield.*—If Dr. Smith will remember, I think Dr. Shaw took in one or two cases of that kind in which he used some such appliance as this bar going around another tooth. I am quite sure he had at least one case in which he used it.

*Dr. Smith.*—I don't remember it.

*Dr. Andrews.*—I would like to ask Dr. Meriam what kind of a file that is, and whether they have them finer cut?

*Dr. Meriam.*—It is called a "crossing" file, and I suppose it is not now used much, as they have machinery for the same work, although they are regularly listed in foreign lists. I think Dr. Lord has had to order them made abroad, and I suppose there would be no difficulty in getting just what you wanted. Abroad they have not the large spirit we have here, and I should say there would be no difficulty in getting a half-dozen files like this, or finer, or coarser, made at a very moderate price.

*Dr. Potter.*—I fell in with Dr. Melotte, of Ithaca, N. Y., this summer, and I was a good deal interested in some things he had to say. One remark he made was this: "If my office was burned down and everything destroyed, and I was about to equip another, the first thing I would buy would be a machine for rolling plate, and I would build up around that my whole outfit." That showed his idea of the importance of being able to work metal.

I learned from Dr. Melotte that he was not in the habit of using solder for uniting the bands of crowns; he brazed the metals together, in order to keep the metal pliable at the joint. His method of changing the shape of thin corundum wheels, such as are used to trim crowns in preparation for fitting bands, was of interest. This was accomplished by revolving the wheel in the engine and holding against the wheel an orange-wood stick. Sufficient heat is developed to soften the wheel and allow its being spun into any desired shape.

*Dr. Werner.*—Do I understand that this process of annealing in a test-tube is original with Dr. Meriam?

*Dr. Meriam.*—I have never heard of it, nor been able to find record of it.

*Dr. Werner.*—Then of course we ought to be and are very glad to know of it, and to have it go on record now. We do not want somebody by and by to patent it and make us pay a royalty on a simple way of annealing steel. If we can anneal, reanneal, temper, and retemper our fine instruments, such as broaches and nerve-canal finders, in this practical way, then Dr. Meriam is entitled to our heartiest thanks for teaching it to us.

*Dr. Gillett.*—If I understand Dr. Meriam rightly, perhaps the main advantage he claims in the manufacture of his own solder is the toughness he gets over solders that he purchases.

*Dr. Meriam.*—I like to know what I am using. I like the elastic quality in solder; when it is somewhat malleable I do not think it is as likely to break. Then I like a great quantity of solder. I do not like a little pennyweight, which you can hardly find, but I like a long sheet from which I can cut off and use as much as I wish. I also like the exact formula by which you get an exactness of color. Another question is the element of expense. I remember, in my last melting of gold I figured up the cost, and there were twelve dollars saved. For instance, my trade with the shops would be about on this basis: you sell your gold scrap at one dollar; any new gold bought from them would be estimated at about one dollar and ten cents.

*Dr. Gillett.*—Do you have your solder made by the same metal worker who makes your plate?

*Dr. Meriam.*—I go to any good refiner and give him my formula, as a physician would a prescription; he follows my directions, and I get exactly what I want. I also take my plate scraps and have those made into plate, but it is not all made according to one formula. There are cases, for instance, where the gum is very much receded, in which I prefer to use a stiffer gold and take more time; that will hold its edge there better than the plate which I use the most of. I use foil scraps for my solder, first having all foreign matter removed, especially the tin. You will notice this smaller crown has a double ring, one ring inside of the other, and the two soldered together; and perhaps you can see how well the solder works there.

*Dr. Werner.*—Are those flexible burnishers which you showed us on the market for sale?

*Dr. Meriam.*—I do not know that they are. Those that I have here were made in the laboratory just a few weeks ago. As I have said, the temper in them was obtained by the use of the hammer. They have never been heated, except for drawing the temper and bending.

*Dr. Werner.*—They seem to be very nice shapes, and we would like to have them go into the trade, the same as some other good things you have given us.

*Dr. Smith.*—Before adjourning, I would like to say that it has been a great pleasure to me, as I know it has to all of you, to hear the voice of Dr. Meriam teaching us—as he always does teach us—something new, and I would move that a vote of thanks be extended to Dr. Meriam for his very interesting remarks this evening.

Unanimously voted.

William H. Potter, D.M.D.,
*Editor American Academy of Dental Science.*

